# Detecting potential causal relationship between multiple risk factors and Alzheimer’s disease using multivariable Mendelian randomization

**DOI:** 10.18632/aging.103983

**Published:** 2020-11-07

**Authors:** Qiang Zhang, Fei Xu, Lianke Wang, Wei-Dong Zhang, Chang-Qing Sun, Hong-Wen Deng

**Affiliations:** 1 School of Nursing and Health, Zhengzhou University, High-Tech Development Zone of States, Zhengzhou 450001, P.R. China; 2 Department of Epidemiology and Biostatistics, College of Public Health, Zhengzhou University, High-Tech Development Zone of States, Zhengzhou 450001, P.R. China; 3 Center for Bioinformatics and Genomics, School of Public Health and Tropical Medicine, Tulane University, New Orleans, LA 70112, USA

**Keywords:** Mendelian randomization, multivariable MR, Alzheimer’s disease, causal relationship

## Abstract

Background: Alzheimer’s disease (AD) is a progressive brain disorder characterized by cognitive skills deterioration that affects many elderly individuals. The identified genetic loci for AD failed to explain the large variability in AD and very few causal factors have been identified so far.

Results: mvMR showed that increasing years of schooling (OR=0.674, 95%CI: 0.571-0.796, *P*=3.337E-06) and genetically elevated HDL cholesterol (OR ranging from 0.697 to 0.830, *P*=6.940E-10) were inversely associated with AD risk, genetically predicted total cholesterol (OR=1.300, 1.196 to 1.412; *P*=6.223E-10) and LDL cholesterol (OR=1.193, 1.097 to 1.296, *P*=3.564E-05) were associated with increasing AD risk. Genetically predicted FG was suggestively associated with increased AD risk. Furthermore, MR-BMA analysis also confirmed FG and years of schooling as two of the top five causal risk factors for AD.

Conclusions: Our findings might provide us novel insights for treatment and intervention into the causal risk factors for AD or AD-related complex diseases.

Methods: By using extension methods of Mendelian randomization (MR)--multivariable MR (mvMR) and MR based on Bayesian model averaging (MR-BMA), we intend to estimate the potential causal relationship between nine risk factors and AD outcome and try to prioritize the most causal risk factors for AD.

## INTRODUCTION

Alzheimer’s disease (AD) is an irreversible, progressive brain disorder characterized by memory, thinking and cognitive skills deterioration that results in behavioral problems among affected elderly individuals. While there are many environmental factors that may influence the risk of AD, it is estimated that nearly 70% of the variance in AD may be explained by genetic determinants [1, 2]. Although GWAS studies have identified over 20 disease-associated genomic loci AD-associated loci, the causal mechanisms implicated in the onset of AD remain unclear [3]. Traditional epidemiology studies reported several common risk factors for AD including obesity [4, 5], type 2 diabetes (T2D) and glycemic traits [6, 7], smoking [8], and lower education level [8]. However, these findings may be influenced by unmeasured confounding factors that may obscure the true relationship.

The Mendelian randomization (MR) approach [9] enables us to assess the potential causal effect of a risk factor on the outcome by using genetic instrumental variables (IVs). Previous two sample MR studies have demonstrated several potential causal risk factors for AD, such as lower education level and smoking [10]. Multivariable MR (mvMR) [11] is an extension of two sample MR approach that incorporates a set of pleiotropic SNPs [12] associated with several risk factors to simultaneously assess the causal effect of each risk factor on the outcome. In comparison with two sample MR, mvMR assumes the genetic IV is associated with at least one risk factor, although not necessarily all risk factors. Additionally, under the circumstances of horizontal pleiotropy, causal effect can be assessed even if none of the variants show specific associations with any individual risk factor [11]. Burgess et al. [13] successfully applied mvMR to estimate the causal effects of lipid fractions on cardiovascular artery disease. However, the current application of mvMR are not capable of feature ranking and selecting.

Recently, Zuber V et al. developed a novel approach [14] which combined multivariable MR with Bayesian model averaging (MR-BMA) that scales to high dimensional settings and can select biomarkers as causal risk factors for the disease of interest. Study demonstrated that the method can detect and prioritize true risk factors even when the multiple risk factors are highly correlated [14]. The MR-BMA approach has been successfully applied to prioritize the most likely causal metabolites for age-related macular degeneration [14].

Of all the previously reported risk factors for AD, it remains unclear which are causal, and which may play the most pivotal role in disease susceptibility. In the current study we intend to integrate the mvMR and MR-BMA approach to identify and prioritize the most likely causal risk factors for AD, the risk factors included in the current study are body mass index (BMI), type 2 diabetes (T2D), high-density lipoprotein cholesterol (HDL cholesterol), low-density lipoprotein cholesterol (LDL cholesterol), total cholesterol, fasting glucose (FG), fasting insulin (FI), currently tobacco smoking, and years of schooling.

## RESULTS

### Genetic IVs selection and validation

Overall, we obtained 1235 LD-independent SNPs that achieved genome-wide significance for all the risk factors after implementing the pruning strategy previously described. Then those SNPs were extracted from AD dataset. After harmonizing the exposure and outcome datasets, there were 1159 SNPs remained for the MR analysis. The number of IVs included for each risk factor was demonstrated in Table 1, and detailed information for the characteristics of SNPs used for each risk factor was shown in Supplementary Table 1.

**Table 1:** Baseline information of exposures and outcomes used in our analysis.

**Exposure**	**Sample size**	**Population**	**PMID**	**Numbers of IVs**
Body mass index (BMI)	461460	European	-	360
Current tobacco smoking	462434	European	-	27
Fasting insulin	108557	European	22885924	13
Fasting glucose	133010	European	22885924	31
HDL cholesterol	196476	Mix*	24097068	85
LDL cholesterol	196476	Mix*	24097068	72
Total cholesterol	196476	Mix*	24097068	78
Type 2 diabetes	659316	European	30054458	101
Years of schooling	1131881	European	30038396	248

**Notes:** Mix population was composed of 188,578 European-ancestry individuals and 7,898 non-European ancestry individuals.

### mvMR estimates results

**Figure 1:**
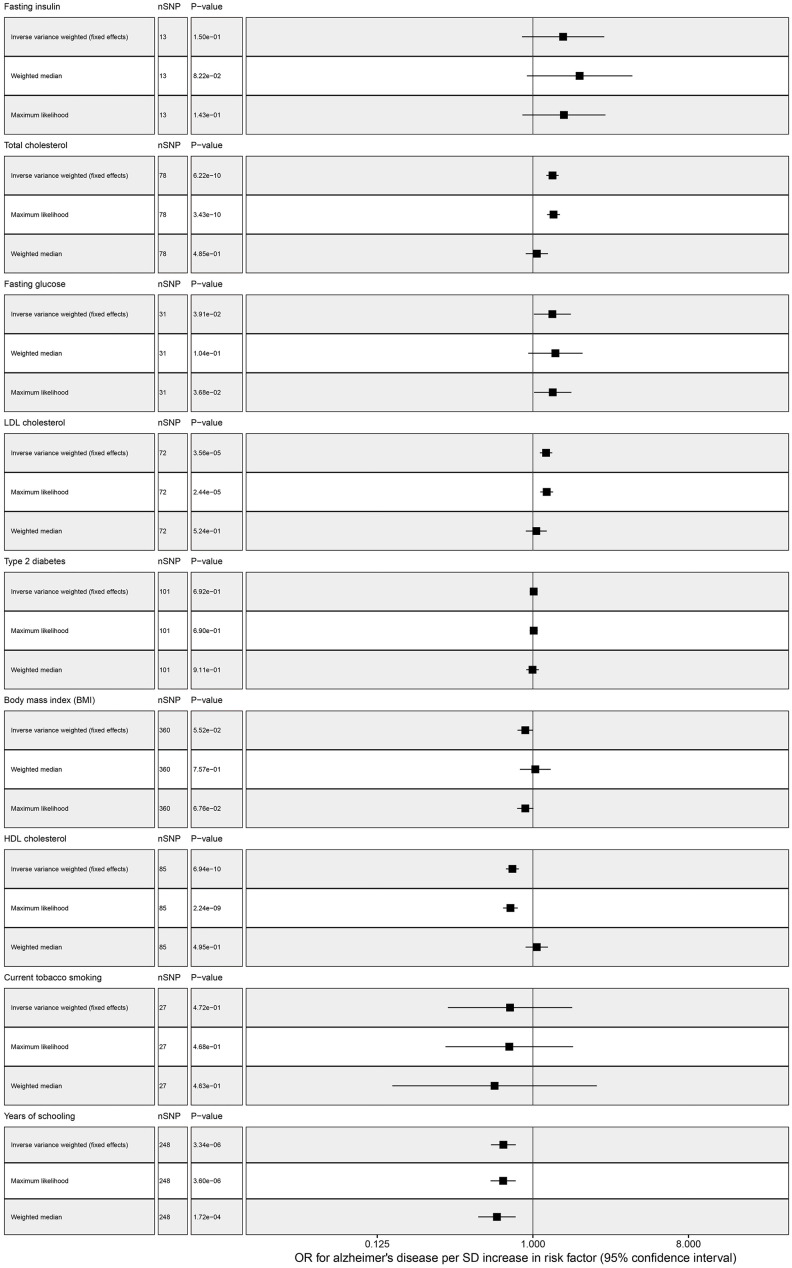
**Multivariable MR analysis forest plot: effect of multiple risk factors on AD.**

Our standard MR approach showed that genetically increased years of schooling (OR = 0.674, 95% confidence interval (CI): 0.571-0.796, *P* = 3.337E-06) and elevated HDL cholesterol (OR = 0.761, 95% CI: 0.697-0.830, *P* = 6.940E-10) were significantly associated with decreasing risk of AD. We found a significant association between total cholesterol and AD, the odds ratio per genetically predicted 1 SD higher total cholesterol level was 1.300 (1.196 to 1.412; *P* = 6.223E-10) (Table 2 and Figure 1). Elevated LDL cholesterol level was associated with increased ADD susceptibility (OR ranging from 1.097 to 1.296 per SD increment in genetically determined LDL cholesterol level, *P* < 3.564E-05). However, there was a suggestive association between FG and AD, one SD increase in FG was associated with 29.7% increase in AD risk (OR = 1.297, 95% CI: 1.013-1.661, *P* = 0.039). No association was observed between the other risk factors and AD, and for detailed information please find Figure 1 and Table 2.

**Table 2:** Multivariable MR analysis for risk factors and AD.

**Exposure**	**Methods**	**nsnp**	**OR**	**OR_lci95**	**OR_uci95**	***P*_value**
Years of schooling	IVW fixed effects	248	0.674	0.571	0.796	**3.337E-06**
Years of schooling	Maximum likelihood	248	0.672	0.568	0.795	**3.597E-06**
Years of schooling	Weighted median	248	0.619	0.481	0.795	**1.814E-04**
T2D	Weighted median	101	0.995	0.915	1.082	9.106E-01
T2D	IVW fixed effects	101	1.011	0.959	1.066	6.921E-01
T2D	Maximum likelihood	101	1.011	0.958	1.067	6.900E-01
Total cholesterol	IVW fixed effects	78	1.300	1.196	1.412	**6.223E-10**
Total cholesterol	Maximum likelihood	78	1.318	1.209	1.436	**3.430E-10**
Total cholesterol	Weighted median	78	1.055	0.912	1.220	4.742E-01
LDL cholesterol	IVW fixed effects	72	1.193	1.097	1.296	**3.564E-05**
LDL cholesterol	Maximum likelihood	72	1.203	1.104	1.311	**2.439E-05**
LDL cholesterol	Weighted median	72	1.047	0.912	1.201	5.151E-01
HDL cholesterol	IVW fixed effects	85	0.761	0.697	0.830	**6.940E-10**
HDL cholesterol	Maximum likelihood	85	0.741	0.671	0.817	**2.243E-09**
HDL cholesterol	Weighted median	85	1.053	0.908	1.222	4.913E-01
Fasting insulin	IVW fixed effects	13	1.498	0.864	2.596	1.497E-01
Fasting insulin	Maximum likelihood	13	1.514	0.869	2.637	1.431E-01
Fasting insulin	Weighted median	13	1.868	0.921	3.787	8.318E-02
Fasting glucose	IVW fixed effects	31	1.297	1.013	1.661	**3.908E-02**
Fasting glucose	Maximum likelihood	31	1.304	1.016	1.673	**3.679E-02**
Fasting glucose	Weighted median	31	1.351	0.929	1.965	1.153E-01
Current tobacco smoking	IVW fixed effects	27	0.737	0.321	1.692	4.717E-01
Current tobacco smoking	Maximum likelihood	27	0.730	0.311	1.711	4.685E-01
Current tobacco smoking	Weighted median	27	0.599	0.161	2.228	4.449E-01
BMI	IVW fixed effects	360	0.904	0.815	1.002	5.524E-02
BMI	Maximum likelihood	360	0.904	0.811	1.007	6.764E-02
BMI	Weighted median	360	1.033	0.842	1.267	7.555E-01

### Sensitivity analysis

Consistent with standard IVW results, MLM and weighted median results also showed a significant association between years of schooling and AD (Table 2). In the analysis of weighted median, genetically determined increasing HDL cholesterol level was associated with decreased AD risk (Table 2). Similar significant association was observed between total cholesterol with AD by MLM approach. Similar to MR main results, MLM approach also found a suggestive association between FG and AD. We still did detect any association between the rest risk factors and AD. Furthermore, MR Egger test suggested that there was no pleiotropic effect among the selected IVs for each risk factor (Table 3).

**Table 3:** Pleiotropic test for the selected instrumental variables.

**Exposure**	**Egger_intercept**	**se**	**pvalue**
Type 2 diabetes	-8.337E-03	0.006	0.186
Years of schooling	4.781E-03	0.005	0.327
HDL cholesterol	2.795E-03	0.019	0.885
LDL cholesterol	-1.158E-02	0.009	0.215
Total cholesterol	-1.663E-02	0.011	0.131
Fasting glucose	5.334E-04	0.009	0.955
Fasting insulin	-4.983E-03	0.026	0.851
Body mass index (BMI)	9.387E-03	0.007	0.202
Current tobacco smoking	6.280E-03	0.019	0.746

For the bi-directional MR analysis, 16 LD independent SNPs that reached genome-wide significance were selected as IVs for AD, then those SNPs were extracted from the nine outcomes individually. After data harmonization, number of valid IVs left for each outcome was demonstrated in Table 4. The results showed significant association between AD and BMI (OR ranging from 0.980 to 0.995, *P* = 0.002), and borderline association between AD and T2D (OR = 1.047, 95 CI: 1.000-1.096, *P* = 0.049, Table 4). And MR Egger intercept suggested no existence of pleiotropic among selected IVs. Besides, as a complementary approach, Steiger test results also showed that the variances explained in the exposuresa were larger than that in the outcome (AD), and the causal direction turned out to be TRUE (Table 5).

**Table 4:** Bi-directional MR results: Alzheimer’s disease as exposure.

**Outcome**	**Methods**	**nsnp**	**OR**	**OR_lci95**	**OR_uci95**	**pvalue**
BMI	IVW fixed effects	13	0.988	0.980	0.995	0.002
BMI	Maximum likelihood	13	0.987	0.979	0.995	0.001
BMI	Weighted median	13	0.988	0.974	1.002	0.093
Current tobacco smoking	IVW fixed effects	13	1.000	0.995	1.004	0.864
Current tobacco smoking	Maximum likelihood	13	1.000	0.995	1.004	0.861
Current tobacco smoking	Weighted median	13	1.000	0.993	1.006	0.889
HDL cholesterol	IVW fixed effects	7	0.984	0.952	1.016	0.324
HDL cholesterol	Maximum likelihood	7	0.983	0.950	1.016	0.312
HDL cholesterol	Weighted median	7	0.983	0.940	1.028	0.454
LDL cholesterol	IVW fixed effects	7	1.001	0.966	1.038	0.949
LDL cholesterol	Maximum likelihood	7	1.001	0.966	1.038	0.948
LDL cholesterol	Weighted median	7	1.014	0.968	1.062	0.567
Total cholesterol	IVW fixed effects	7	0.988	0.954	1.023	0.489
Total cholesterol	Maximum likelihood	7	0.988	0.953	1.023	0.489
Total cholesterol	Weighted median	7	0.987	0.945	1.031	0.557
Type 2 diabetes	IVW fixed effects	7	1.047	1.000	1.096	0.049
Type 2 diabetes	Maximum likelihood	7	1.049	1.001	1.099	0.044
Type 2 diabetes	Weighted median	7	1.011	0.948	1.078	0.735
Years of schooling	IVW fixed effects	13	0.999	0.992	1.006	0.828
Years of schooling	Maximum likelihood	13	0.999	0.992	1.006	0.821
Years of schooling	Weighted median	13	1.008	0.999	1.017	0.090

**Table 5:** MR Steiger directionality test results.

**Exposure**	**snp_r2.exposure**	**snp_r2.outcome**	**Correct_causal_direction**	**Steiger_pvalue**
Type 2 diabetes	0.124	0.003	TRUE	0
Years of schooling	0.019	0.007	TRUE	2.09E-34
HDL cholesterol	0.053	0.020	TRUE	1.96E-79
LDL cholesterol	0.058	0.008	TRUE	4.38E-212
Total cholesterol	0.064	0.013	TRUE	2.09E-191
Fasting glucose	0.032	0.001	TRUE	2.13E-182
Fasting insulin	0.005	0.0002	TRUE	1.81E-26
Body mass index (BMI)	0.058	0.027	TRUE	7.22E-71
Current tobacco smoking	0.003	0.001	TRUE	9.74E-06

### MR-BMA estimates results

All the risk factors were then prioritized and ranked by their MIP. The top five risk factors for AD were current tobacco smoking (MIP = 0.289), BMI (MIP = 0.210), FI (MIP = 0.164), FG (MIP = 0.163), years of schooling (MIP = 0.161), and which were confirmed to be included in the best five individual models with their PP values of 0.207, 0.138, 0.107, 0.112 and 0.111 respectively (Table 6). The MR-BMA were partially consistent with mvMR results, MR-BMA method prioritized two of the significant risk factors (FG and years of schooling) in mvMR result.

**Table 6:** Ranking of risk factors for AD. A) According to their marginal inclusion probability (MIP).

	**Risk factors combination**	**Marginal inclusion probability (MIP)**	**Model averaged causal estimate**
**1**	Current tobacco smoking	0.289	8.582E-02
**2**	BMI	0.210	-4.085E-02
**3**	Fasting insulin	0.164	2.502E-02
**4**	Fasting glucose	0.163	3.087E-02
**5**	Years of schooling	0.161	1.195E-02
**6**	LDL cholesterol	0.093	9.312E-03
**7**	Total cholesterol	0.062	4.005E-03
**8**	HDL cholesterol	0.036	-5.559E-04
**9**	T2D	0.024	3.122E-04

**B):** The best ten individual models according to their posterior probability (PP).

**Individual models**	**Risk factors combination**	**Posterior probability**
**9**	Current tobacco smoking	0.207
**8**	BMI	0.138
**6**	Fasting glucose	0.112
**2**	Years of schooling	0.111
**7**	Fasting insulin	0.107
**4**	LDL cholesterol	0.062
**5**	Total cholesterol	0.041
**3**	HDL cholesterol	0.024
**8,9**	BMI, current tobacco smoking	0.018
**1**	T2D	0.015

## DISCUSSION

In the present study, by performing mvMR and MR-BMA analysis together using summary statistics for AD and multiple risk factors, we successfully identified five causal risk factor (years of schooling, total cholesterol, HDL cholesterol, LDL cholesterol and FG) for AD and we also prioritized and ranked two of these five risk factors (FG and years of schooling) for AD, which might provide us novel insights into determine the causal risk factors for complex traits and diseases.

Our results are consistent with both previous traditional observational studies and two sample MR results which provide established evidence that educational attainment was associated with a reduced risk of AD [21–23]. Concentrations of genetically determined total cholesterol and LDL cholesterol showed positive associations with risks of AD, which is consistent with the known causal effect of them on AD risk from previous two sample MR study [24], and similar to our results, elevated HDL cholesterol level also showed causal association with decreased AD risk [24]. The suggestive association between FG and higher AD risk is also consistent with previous study that FG showed suggestive association with AD risk in sensitivity analysis [24]. No observed causal associations between fasting insulin, BMI and AD risk are also consistent with previous two sample MR result [24].

Although our mvMR did not find any causal association between current tobacco smoking with risk of AD, current tobacco smoking became the top first risk factor in the MR-BMA model, which is partially in accordance with previous MR study [24].

However, the observed associations differ from previous study that no evidence of causal association between lipid profiles and AD risk after excluding the potential pleiotropic SNPs. In the current study, the independent SNPs (r^2^ < 0.001) we included suggest no evidence of pleiotropic effect and our sensitivity analysis support the causal associations detected by the main MR analysis. One of the most interesting findings from the present study is the absence causal association of BMI and AD risk, however the bi-directional MR found causal association between AD and decreased BMI. This finding supports the hypothesis of reverse causation (the negative confounding effect of AD related weight loss) might be an explanation for the obesity paradox on AD risk [4].

There are several important strengths to note for the current study. Our MR analysis results may provide evidence of the causal role of five risk factors in the development of AD since the influence of traditional confounding factors in observational studies is minimized/eliminated. Since the alleles follow the principle of random distribution when forming gametes at meiosis, the causal effect of genotype on disease in MR studies will not be distorted by the confounding factors, a major limitation of traditional observational studies. By leveraging the summary statistics from the large available GWASs for multiple risk factors and AD, we were able to increase our discovery power. Furthermore, previous studies have shown that performing the MR analysis by using summary statistics data and by using individual-level data have similar efficiency [19]. Finally, the application of MR-BMA ranked the potential risk factors for AD, which could provide certain genetic evidence in disease prevention and curation.

However, there may also be some limitations. First, we included the mixed population data for lipid traits instead of European only, because the dataset for lipid we included in our analysis was the largest to date, and the individuals from other ethnicities only take up 4% (Table 1). Additionally, our MR results does not mean the potentially causal risk factors identified are playing truly causal roles in the AD susceptibility, we were trying to provide some novel insights into underlying mechanisms of the AD and hope to provide certain genetical evidence to disease prevention.

## CONCLUSIONS

In conclusion, by combining mvMR and MR-BMA together, we successfully identified five potential causal risk factors for AD and we also ranked and prioritized two of them for AD, which might provide us novel insights into the causal mechanisms of AD. Our results demonstrate that by increasing the years of schooling and HDL cholesterol level, decreasing total cholesterol, LDL cholesterol and FG levels could decrease the risk of developing AD.

## MATERIALS AND METHODS

### Genetic IVs selection and validation

Summary statistics for risk factor-associated SNPs were extracted from the large publicly available GWAS datasets to date performed by the corresponding Consortia in European populations (Table 1). For the implementation of mvMR, we selected SNPs that achieved genome-wide significance (p < 5 × 10^−8^) in the GWAS datasets as for each risk factor as IVs. Effect estimates of these risk factor-associated SNPs on the risk of AD were assessed using the summary statistics of 74,046 European individuals for AD from The International Genomics of Alzheimer's Project (IGAP) Consortium [15]. The European samples from the 1000 genomes project reference panel were adopted to estimate linkage disequilibrium (LD) between chosen SNPs. When target SNPs were not available in the outcome study, we used proxy SNPs that were in high LD (r^2^ > 0.8) with the SNPs of interest.

To ensure the SNPs used as IVs for risk factors are not in LD with each other, a vital assumption of MR, we calculated pairwise-LD between all our selected SNPs in the 1000 Genomes European reference sample using PLINK 1.90 [16]. For all pairs of SNPs determined to violate the independence assumption with r^2^ > 0.001 we retained only the SNP with the smaller association p-value. To ensure the effect of a SNP on the exposure and the effect of that SNP on the outcome correspond to the same allele, we harmonized the effect of these instrumental SNPs by using a function that ensures all corresponding risk factors and outcome (AD) alleles are on the same strand where possible. If they are not, then the function will flip alleles and use allele frequency to infer the strand of palindromic SNPs.

### mvMR estimates

In the current study, standard inverse-variance-weighted (IVW) fixed-effects [17] analysis was used to estimate the causal effect of the multiple related risk factors on the BMD traits. After obtaining the selected instruments for each exposure, all exposures for those SNPs were then regressed against the outcome together, weighting for the inverse variance of the outcome to ensure the genetic instruments with more precise association receive more weight in our analysis.

### Sensitivity analysis

As sensitivity analysis, weighted median estimator and maximum likelihood method (MLM) [18] were also performed to provide more robust MR estimates. MR-Egger approach [19] was also used to assess the potential pleiotropic effects among the selected IVs. A Bonferroni corrected threshold with *P* < 0.006 (0.05/9) was considered to be significant causal association, and 0.006 < P < 0.05 was considered suggestive evidence for causal association. Besides, to orient the causal relationship between them, we also performed the bi-directional MR analysis (*P* < 0.05) and MR Steiger directionality test [20].

### MR-BMA estimates

Following the mvMR analysis, MR-BMA was applied to prioritize the most causally related risk factors for AD. MR-BMA assumes that the true causal risk factors are very few and it considers the risk factor selection as a variable selection problem in the linear regression model. The approach considers all possible combinations of the risk factors and generates posterior probability (PP) for each specific model, where PP means the probability of including a specific risk factor in the model. Furthermore, MR-BMA adopts BMA which computes a marginal inclusion probability (MIP) for each risk factor, where MIP refers to the sum of the PP over all possible models where the risk factor is present. Then MR-BMA will compute the model-averaged causal estimate (MACE) for each risk factor by ranking all the risk factors according to the corresponding MIP. Finally, MR-BMA will prioritize the best model by the PP value for each individual model. All the analyses were implemented in R software environment.

### Availability of data and materials

The datasets generated and/or analyzed during the current study are included in this published article and provided in Supplementary Table 1 of Supplementary Materials.

## Supplementary Material

Supplementary Table 1
